# Association between antiepileptic drugs and hepatocellular carcinoma in patients with epilepsy: a population‐based case–control study

**DOI:** 10.1002/brb3.554

**Published:** 2016-09-12

**Authors:** Dong‐Zong Hung, Cheng‐Li Lin, Yi‐Wen Li, Yen‐Ning Lin, Ying‐Ray Lee, Charles‐C. N. Wang, Jih‐Jung Chen, Yun‐Ping Lim

**Affiliations:** ^1^ Graduate Institute of Clinical Medical ScienceCollege of MedicineChina Medical UniversityTaichungTaiwan; ^2^ Department of EmergencyToxicology CenterChina Medical University HospitalTaichungTaiwan; ^3^ Management Office for Health DataChina Medical University HospitalTaichungTaiwan; ^4^ School of MedicineChina Medical UniversityTaichungTaiwan; ^5^ Department of PharmacyCollege of PharmacyChina Medical UniversityTaichungTaiwan; ^6^ Translational Medicine Research CenterChia‐Yi Christian HospitalChiayiTaiwan; ^7^ Department of Biomedical InformaticsAsia UniversityTaichungTaiwan; ^8^ Graduate Institute of Pharmaceutical TechnologyTajen UniversityPingtungTaiwan

**Keywords:** antiepileptic drugs, case–control study, epilepsy, hepatocellular carcinoma, population‐based

## Abstract

**Background:**

This study explored whether antiepileptic drugs (AEDs) use increases the risk of hepatocellular carcinoma (HCC).

**Methods:**

We conducted a case–control study using data from the National Health Insurance system of Taiwan. The case group comprised 1,454 epilepsy patients with newly diagnosed HCC, and the control group comprised 1,448 epilepsy patients without HCC. Both groups had similar distributions of sex and age, and follow‐up duration. Possible associations with the AEDs in Taiwan were examined.

**Results:**

After adjusted for AEDs (phenobarbital and primidone, clonazepam, clorazepate and diazepam, and other AEDs), and for the comorbidities of diabetes, chronic liver disease and cirrhosis, hepatitis B and C virus infection, and alcoholism, the odds ratio (OR) of HCC was 1.22 (95% confidence interval [CI]: 1.01–1.47) for the group of phenytoin users compared with nonphenytoin users. An annual means of 61–120, 121–180, and >180 of defined daily doses (DDDs) of phenytoin (OR: 4.07, 95% CI: 2.03–8.18; OR: 7.51, 95% CI: 3.03–18.7, and OR: 14.6, 95% CI: 7.88–26.9, respectively) were significantly correlated with the risk of HCC but not with a DDD of ≤60. Compared with nonphenytoin users, HCC patients who had used phenytoin within 1 year of HCC diagnosis were at a greatest risk of HCC (adjusted OR: 2.29, 95% CI: 1.71–3.08), followed by who had used phenytoin within 2 years of diagnosis (adjusted OR: 1.92, 95% CI: 1.44–2.56).

**Conclusion:**

The results indicate that high dose of phenytoin was associated with a statistically significant increased OR for HCC, which was not demonstrated for low‐dose phenytoin.

## 1 Introduction

Epileptic seizures are produced by abnormal discharges of neurons that may be caused by any pathological process affecting the brain (Devinsky, Gazzola, & LaFrance, 2011). Epileptic seizures are common. The incidence rate was estimated to be between 20 and 70 cases per 100,000 persons, and the cumulative incidence was estimated to be 2%–5% (Chang & Lowenstein, 2003). According to the classifications by the American Hospital Formulary Service Drug Information (AHFS/DI), several categories of antiepileptic drugs (AEDs) are commercially available in Taiwan, including barbiturates (phenobarbital and primidone), clonazepam, hydantoins (phenytoin), benzodiazepines (clorazepate and diazepam), and other AEDs (e.g., carbamazepine, gabapentin, lamotrigine, and levetiracetam). Several routinely used AEDs are ranked as the top causes of clinically apparent drug‐induced liver injury (Björnsson, 2008; Imam et al., 2014; Kazamatsuri, 1970; Metreau, Bismuth, Franco, Franco, & Dhumeaux, 1975; Reuben, Koch, Lee, & Acute Liver Failure Study Group, 2010; Sato et al., 2005; Verrotti, Di Marco, la Torre, Pelliccia, & Chiarelli, 2009; vanZoelen et al., 2012).

Cancer remains a leading cause of morbidity and mortality worldwide. In 2012, nearly 14 million new cases arose and 8.2 million cancer‐related deaths occurred (Stewart & Wild, 2014). Cancer can complicate numerous chronic diseases, including epilepsy (Singh, Driever, & Sander, 2005). AEDs are often used over long periods of time, which may increase the tendency for some adverse effects, including carcinogenesis. The purpose of this study was to determine whether there was an association between any specific AED and hepatocellular carcinoma (HCC) in patients with epilepsy in Taiwan. In 2014, HCC was the second leading cause of cancer deaths (after lung cancer) in Taiwan, with a mortality rate of 34.9 per 100,000 people (18.6% of the total cases) (Department of Health 2012). Because of the importance of epilepsy treatment, the potential of AEDs to cause hepatotoxicity must be considered.

To the best of our knowledge, no large‐scale case–control study to date has addressed the association between HCC and the use of AEDs in Taiwan. Thus, we conducted a population‐based case–control study using data from the National Health Insurance (NHI) program of Taiwan to determine the association between AEDs and the risk of HCC.

## 2 Materials and Methods

### 2.1 Data sources

Taiwan's universal NHI program has been in operation since 1995, and nearly 100% of all residents are enrolled. The National Health Research Institutes (NHRI) was commissioned to construct and maintain the National Health Insurance Research Database (NHIRD) for researchers. The NHIRD provides detailed information on the healthcare services used by each patient, as well as the demographic characteristics, complete outpatient visits, hospital admissions, International Classification of Diseases, Ninth Revision, Clinical Modification (ICD‐9‐CM) diagnostic codes, prescriptions, and clinical orders (such as surgery) of participants and utilities. The NHRI encrypts patients’ personal information for privacy protection and provides researchers with anonymous identification numbers associated with the relevant claims information. This study was approved by the Institutional Review Board of China Medical University Hospital (CMUH104‐REC2‐115).

### 2.2 Study patients

**Figure 1:**
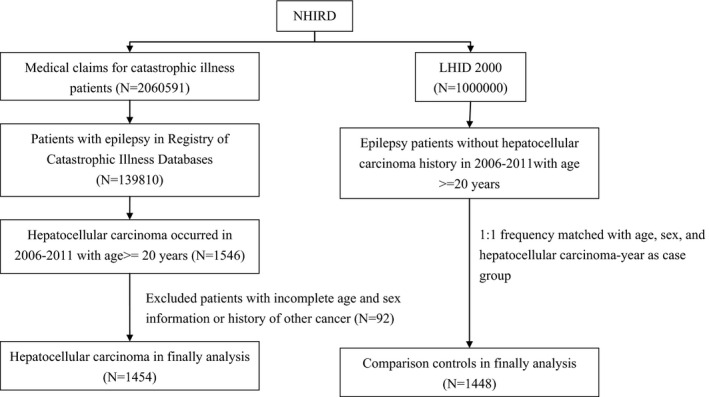
Flowchart of participant recruitment in this study

Our research was based on case–control study patients with epilepsy (ICD‐9‐CM code 345) who were indentified using two subdatasets of the NHIRD, medical claims data from the Catastrophic Illness Patients Database and Longitudinal Health Insurance Database 2000 (LHID 2000) (Fig. 1). We first identified patients with epilepsy in the Registry of Catastrophic Illness Patient Databases, which contains health claims data for the treatment of catastrophic illnesses, including 30 categories of diseases that require long‐term care. Patients with a catastrophic illness certification who receive care for the illness or related conditions are exempt from copayments for outpatient or inpatient care. We selected patients newly diagnosed with HCC (ICD‐9‐CM code 155) in the period 2006–2011 and aged at least 20 years. The date of HCC diagnosis was defined as the index date. Control participants with epilepsy and without HCC (the non‐HCC group) were identified from the LHID 2000, which comprises 1 million randomly sampled beneficiaries from the NHIRD. There were no significant differences in sex, age, and healthcare costs between LHID 2000 and all insurants in the NHIRD. Each HCC case was selected 1:1 frequency matched with a non‐HCC control with regard to sex, age (in 5‐year bands), and the year of HCC diagnosis. Patients in both groups with a history of other cancers (ICD‐9‐CM codes 140–208) or for whom it was impossible to obtain a complete dataset, were excluded from the study.

A diagnosis of HCC was validated using imaging evidence (such as abdominal ultrasound, angiogram, or CT) supported with other clinical and serological data, and by histopathological confirmation. All insurance claims were scrutinized by medical reimbursement specialists and peer reviewed, according to the standard diagnosis criteria for HCC as described in ICD‐9‐CM; wrong diagnoses or coding errors made by the doctors or hospitals involved, would be heavily penalized. Therefore, the diagnoses of HCC, and the ICD‐9 codes, in this study were highly reliable.

### 2.3 AEDs exposure and comorbidities

We conducted a comprehensive review and classified several categories of commercially available AEDs in Taiwan, according to the AHFS/DI codes, including barbiturates (phenobarbital and primidone), clonazepam, hydantoins (phenytoin), benzodiazepines (clorazepate and diazepam), and other AEDs (including carbamazepine, gabapentin, lamotrigine, levetiracetam, oxcarbazepine, pregabalin, tiagabine, topiramate, valproate, and zonisamide) were selected for analysis. Medication use records were retrieved from ambulatory and inpatient claims data. The accumulated defined daily doses (DDDs) prescribed during the study period were calculated for each patient. The DDD is a statistical measure of drug consumption defined by the World Health Organization as “the assumed average maintenance dose per day for a drug used for its main indication in adults.” It is used to standardize the quantities of drugs administered, and thus, facilitate comparison among drugs. The accumulated DDDs used in this study were the total accumulated doses of phenytoin (ATC code, N03AB02) used by each patient before the diagnosis of HCC converted to DDDs. According to the total supply in days and the accumulated DDDs of phenytoin, we calculated the annual mean DDD of phenytoin for phenytoin users. The annual mean DDD was further partitioned into four levels. For each participant, the records of comorbidities before the index date were obtained using the following codes: diabetes (ICD‐9‐CM code 250), chronic liver disease and cirrhosis (ICD‐9‐CM code 571), hepatitis B virus (HBV) infection (ICD‐9‐CM codes V02.61, 070.20, 070.22, 070.30, and 070.32), hepatitis C virus (HCV) infection (ICD‐9‐CM codes V02.62, 070.41, 070.44, 070.51, and 070.54), and alcoholism (ICD‐9‐CM codes 291, 303, 305.00, 305.01, 305.02, 305.03, 790.3, and V11.3).

### 2.4 Statistical analysis

The distributions of sex, age (20–39, 40–64, 65–74, and ≥75 years), AED medications, and baseline comorbidities were compared between the HCC group and the non‐HCC group, and were examined using the chi‐square test for categorical variables and the Student's *t* test for continuous variables. Univariate and multivariate logistic regression models were used to calculate the odds ratio (OR) and 95% confidence intervals (CIs) for the association between HCC and AEDs use. The multivariate analysis was performed to adjust for the AEDs of phenobarbital and primidone, clonazepam, clorazepate and diazepam, and other AEDs, and the comorbidities of diabetes, chronic liver disease and cirrhosis, HBV infection, HCV infection, and alcoholism. We also estimated the risk of HCC by cumulative dose for phenytoin use. Furthermore, we analyzed the association between HCC and the time difference between the last phenytoin in use and the index date. All analyses were performed using SAS statistical software for Windows (Version 9.4; SAS Institute Inc., Cary, NC, USA), and the significance level was set at .05.

## 3 Results

The case group comprised 1,454 epilepsy patients with newly diagnosed HCC, and the control group comprised 1,448 epilepsy patients without HCC. Both groups had similar distributions of sex and age, and were predominantly male (73.5%). Approximately, 40% were older than 75 years of age. The mean ages of the HCC patients and non‐HCC controls were 64.9 ± 13.6 and 64.7 ± 13.7 years, respectively. The HCC group exhibited a higher prevalence of all baseline comorbidities than did the non‐HCC control patients (Table 1). Table 2 shows the crude and adjusted ORs for the model fitted to examine the association between AEDs use and the development of HCC. The adjusted OR for HCC risk in patients who received phenytoin compared with those who did not was 1.22 (95% CI: 1.01–1.47). The risk of HCC increased significantly with the comorbidities of diabetes, chronic liver disease and cirrhosis, HBV infection, HCV infection, and alcoholism.

**Table 1:** Baseline characteristics of the patients

	Hepatocellular carcinoma				*p* value^a^
	No*N* = 1,448		Yes*N* = 1,454		
	*N*	%	*n*	%	
Gender					
Women	384	26.5	385	26.5	.98
Men	1,064	73.5	1,069	73.5	
Age group (year)					
20–39	209	14.4	209	14.4	.99
40–64	325	22.4	324	22.3	
65–74	334	23.1	335	23.0	
≥75	580	40.1	586	40.3	
Mean (*SD*) (year)^a^	64.7	13.7	64.9	13.6	.62
Medications					
Phenobarbital and primidone	12	0.83	9	0.62	.51
Clonazepam	552	38.1	566	38.9	.66
Phenytoin	694	47.9	750	51.6	.04
Clorazepate and diazepam	1,189	82.1	1,264	86.9	<.001
Others^b^	688	47.5	771	53.0	.003
Baseline comorbidities					
Diabetes	321	22.2	454	31.2	<.001
Chronic liver disease and cirrhosis	777	53.7	1,325	91.1	<.001
Hepatitis B virus infection	82	5.66	538	37.0	<.001
Hepatitis C virus infection	46	3.18	547	37.6	<.001
Alcoholism	133	9.19	250	17.2	<.001

Data are presented as the number of participants in each group, with percentages given in parentheses.

^a^Chi‐square test and *t* test comparing participants with and without hepatocellular carcinoma.

^b^Other AEDs including carbamazepine, gabapentin, lamotrigine, levetiracetam, oxcarbazepine, pregabalin, tiagabine, topiramate, valproate, and zonisamide.

**Table 2:** Odds ratios (OR) and 95% confidence intervals (CI) for hepatocellular carcinoma associated with phenytoin and covariates

Variable	Crude		Adjusted^a^	
	OR	95% CI	OR	95% CI
Medications				
Phenobarbital and primidone	0.75	0.31, 1.78	1.27	0.44, 3.66
Clonazepam	1.04	0.89, 1.20	0.93	0.76, 1.13
Phenytoin	1.16	1.00, 1.34*	1.22	1.01, 1.47*
Clorazepate and diazepam	1.45	1.18, 1.78***	0.92	0.71, 1.20
Others^b^	1.25	1.08, 1.44**	1.07	0.88, 1.30
Baseline comorbidities				
Diabetes	1.59	1.35, 1.88***	1.51	1.22, 1.87***
Chronic liver disease and cirrhosis	8.87	7.20, 10.9***	3.46	2.73, 4.38***
Hepatitis B virus infection	9.78	7.64, 12.5***	9.10	6.97, 11.9***
Hepatitis C virus infection	18.4	13.5, 25.1***	16.8	12.1, 23.4***
Alcoholism	2.05	1.64, 2.57***	1.84	1.40, 2.41***

^a^Adjusted for the epilepsy drugs of phenobarbital and primidone, clonazepam, clorazepate and diazepam, and other AEDs, and for the comorbidities of diabetes, chronic liver disease and cirrhosis, hepatitis B virus infection, hepatitis C virus infection, and alcoholism.

^b^Other AEDs including carbamazepine, gabapentin, lamotrigine, levetiracetam, oxcarbazepine, pregabalin, tiagabine, topiramate, valproate, and zonisamide.

*p <* *.05; **p <* *.01; ***p <* *.001.

In this study, the association between HCC risk and the annual mean DDD of phenytoin were analyzed for the period that patients were undergoing monotherapy. The association between HCC risk and the annual mean DDD of phenytoin use is shown in Table 3. Compared with nonphenytoin users, the HCC risk was highest in patients who were administered >180 annual mean DDDs of phenytoin (adjusted OR: 14.6, 95% CI: 7.88–26.9); and was found an OR of 7.51 and 4.07 (95% CI: 3.03–18.7; 2.03–8.18, respectively) in group of annual mean DDDs of 121–180 and 61–120, respectively; however, the risk of HCC among those with a ≤60 annual mean DDDs of phenytoin was not statistically significant. In addition, as this study has been conducted in people with epilepsy, by including the epilepsy status, we analyzed the association between HCC and the time difference among the last phenytoin use and the index date. Compared with the nonphenytoin users, the HCC patients, who last used phenytoin within 1 year prior to the index date, were at a higher risk of HCC (adjusted OR: 2.29, 95% CI: 1.71–3.08), than patients who last used phenytoin within 2 years prior to the index date (adjusted OR: 1.92, 95% CI: 1.44–2.56) (Table 4).

**Table 3:** Odds ratios (OR) and 95% confidence intervals (CI) for hepatocellular carcinoma associated with annual mean defined daily doses of phenytoin

	Case number/control number	Crude OR	95% CI	Adjusted OR^a^	95% CI
Nonuse of phenytoin^b^	704/754	1.00	Reference	1.00	Reference
Phenytoin					
≤60 DDD	508/659	0.83	0.71, 0.96***	0.83	0.68, 1.02
61–120 DDD	56/14	4.28	2.36, 7.76***	4.07	2.03, 8.18***
121–180 DDD	49/7	7.50	3.37, 16.7***	7.51	3.03, 18.7***
>180 DDD	137/14	10.5	5.99, 18.3***	14.6	7.88, 26.9***
*p* for trend					<.001

^a^Adjusted for the epilepsy drugs of phenobarbital and primidone, clonazepam, clorazepate and diazepam, and other AEDs, and for the comorbidities of diabetes, chronic liver disease and cirrhosis, hepatitis B virus infection, hepatitis C virus infection, and alcoholism.

^b^Other AEDs including carbamazepine, gabapentin, lamotrigine, levetiracetam, oxcarbazepine, pregabalin, tiagabine, topiramate, valproate, and zonisamide.

*p <* *.05; **p <* *.01; ***p <* *.001.

**Table 4:** Odds ratios for hepatocellular carcinoma for the phenytoin and nonphenytoin groups

Time for phenytoin use	Non‐HCC		HCC		Adjusted odds ratio^a^	95% CI
	Phenytoin no.	%	Phenytoin no.	%		
Nonuse of phenytoin^b^	754	52.1	704	48.4	1.00	Reference
Within 1 year prior to index	135	15.2	272	27.9	2.29	1.71, 3.08***
Within 2 year prior to index	156	17.1	248	26.1	1.92	1.44, 2.56***

^a^Adjusted for the AEDs of phenobarbital and primidone, clonazepam, clorazepate and diazepam, and other AEDs, and for the comorbidities of diabetes, chronic liver disease and cirrhosis, hepatitis B virus infection, hepatitis C virus infection, and alcoholism.

^b^Other AEDs including carbamazepine, gabapentin, lamotrigine, levetiracetam, oxcarbazepine, pregabalin, tiagabine, topiramate, valproate, and zonisamide.

*p <* *.05; **p <* *.01; ***p <* *.001.

## 4 Discussion

We conducted a comprehensive population‐based case–control study, using the NHIRD to investigate the association between AEDs and the risk of HCC in a group of 2,902 epilepsy patients. The analyses of the HCC group (1,454 patients) and the non‐HCC group (1,448 patients) were adjusted for baseline comorbidities, comprising diabetes, chronic liver disease and cirrhosis, HBV and HCV infection, and alcoholism, all of which might be risk factors for HCC. After adjusted for these comorbidities and age, sex, phenobarbital and primidone, clonazepam, clorazepate and diazepam, and other AEDs (carbamazepine, gabapentin, lamotrigine, levetiracetam, oxcarbazepine, pregabalin, tiagabine, topiramate, valproate, and zonisamide), phenytoin users were still more likely to be diagnosed with HCC (OR: 1.22, 95% CI: 1.01–1.47, *p *<* *.05). In addition, participants with the highest dose of phenytoin, those with an annual mean DDD >180, had an OR of 14.6 (*p *<* *.001), and as such were significantly associated with the increased risk of HCC. Furthermore, the association between the increased risk of HCC and the time difference between the last phenytoin use and the index date, compared with nonphenytoin users, showed that the HCC patients with a last phenytoin use within 1 year prior to the index date were at the greatest risk (adjusted OR: 2.29, 95% CI: 1.71–3.08). The adjusted OR of patients with a last phenytoin use within 2 years prior to the index date was 1.92 (95% CI: 1.44–2.56). Research from Kaae, Carstensen, Wohlfahrt, Melbye, and Allison (2014) also indicated a higher risk of cancer in the first year after the start of AEDs treatment.

Use of specific AEDs is based on several different factors, including patient age, sex, epileptic syndromes, and comorbid diseases. We used the Taiwan NHIRD, which follows the diagnostic coding system recommended by the ICD‐9‐CM; according to the criteria of ICD‐9‐CM, there are separate codes indicating epilepsy and actively treated epilepsy (defined as use of an AED more than once). In Taiwan, 71% of patients were prescribed monotherapy (Hsieh & Huang, 2009), and it is reported that, assuming appropriate choice of monotherapy for newly diagnosed epilepsy, 75% of cases will be free from seizures (Mattson et al., 1985). Monotherapy is reported to be the gold standard for the treatment of epilepsy as it is effective in nearly 80% of patients (Deckers, 2002). Carbamazepine, phenytoin, valproic acid are the most common choices for initial monotherapy in Taiwan (Hsieh & Huang, 2009).

The epidemiological studies on the effect of the use of epilepsy medication on the increase of cancer risk have yielded conflicting results. In the study by Lamminpää, Pukkala, Teppo, and Neuvonen (2002), a cohort of 14,487 male and 13,932 female patients were included, during the follow‐up period, over 40% of the them was attributed to brain cancer and nervous system caner. However, there was no significant incidence difference in any other cancer, including liver cancer. In addition, some authors observed a strong association between epilepsy and the rate of cancer of the CNS and of the mouth and throat (IRRs 2.00–3.91), and a small to moderate association between epilepsy and the prevalence of digestive organs and respiratory tract cancers (IRRs 1.17–1.35), independent of AEDs use. There was no association between epilepsy and the other specified forms of cancer (including liver) in patients with no record of AEDs use. The risk of CNS cancers almost doubled (IRRs 3.91 vs. 2.00) in epilepsy patients treated with AEDs in comparison with that in epilepsy patients not treated with AEDs. They also found that, an increased risk of liver cancer is associated with epilepsy in people using AEDs (Kaae et al., 2014). A follow‐up study from Olsen et al., found that epilepsy patients were at significantly increased risks of brain and CNS (RR = 5.7) as well as lung (RR = 1.4) cancer. However, the incidence of bladder cancer and malignant melanoma significantly decreased (RR = 0.6 vs. 0.5). The risk of non‐Hodgkin's lymphoma increased but the change was not significant (RR = 1.4), this may have been associated with the use of phenytoin (Olsen, Boice, Jensen, & Fraumeni, 1989). Another nested case–control study revealed a significantly high risk of lung cancer but a low risk of bladder cancer among 8,004 epileptic patients in Denmark (Olsen et al., 1993). However, lung cancer was not associated with any AEDs, and the use of phenobarbital was inversely correlated with bladder cancer (Olsen et al., 1993).

Previous studies using phenobarbital and phenytoin in a rodent model revealed that phenobarbital caused liver tumors, whereas phenytoin promoted not only liver tumors but also lymphoma (Braeuning et al., 2014; Diwan, Henneman, Nims, & Rice, 1993; Murray, Hill, Hegemier, & Hurwitz, 1996). Although phenobarbital represented a potent nongenotoxic liver tumor promoter in rodents, the role of phenobarbital in the cancer risk of epilepsy patients remains controversial (La Vecchia & Negri, 2014). Phenytoin has been implicated in the causation of human lymphoma, myeloma, and neuroblastoma (Tittle & Schaumann, 1992). Evidence of carcinogenicity from long‐term pharmacoepidemiological data on these two types of AEDs is inconsistent. Our results did not show a correlation between phenobarbital and HCC; however, we observed that phenytoin was associated with the risk of HCC, particularly in the patients with the DDD of >60.

Valproate has been reported to have an antiproliferative effect in some cancers, both in vitro and in vivo (Abaza, Bahman, & Al‐Attiyah, 2014; Wang et al., 2013). A multicenter, randomized Phase II study was conducted to combine weekly paclitaxel medication with valproate as second‐line chemotherapy to improve survival in gastric cancer patients (Fushida et al., 2015). Recently, new AEDs appear to be safe; however, there is no regulatory testing to monitor carcinogenicity after listing.

Phenytoin (5,S‐diphenylhydantoin or dilantin), which was introduced into medicine in 1938, may cause benign lymph adenopathy and malignant lymphoma, according to some reports (Murray et al., 1996; Sharma, Menon, Sengar, & Gujral, 2013). An experimental rodent study also found that phenytoin treatment led to lymphoma through an immunosuppressive effect and chronic antigenic stimulation (Dethloff, Graziano, Goldenthal, Gough, & de la Iglesia, 1996). However, not all studies have indicated the potentially carcinogenic effect of phenytoin (Lamminpää et al., 2002; Maeda et al., 1988). Initial reports described the use of phenytoin associated with lymphoma after prolonged exposure (Abbondazo, Irey, & Frizzera, 1995; Gleichmann, Pals, Radaszkiewicz, & Wasser, 1982; Johns, Moscinski, & Sokol, 2010). Some reports have described the incidence of neuroblastoma among children with fetal hydantoin syndrome following prenatal exposure to phenytoin (al‐Shammri, Guberman, & Hsu, 1992; Satgé, Sasco, & Little, 1998). Liver cancer (87% incidence) and hepatoblastoma (33% incidence) were found with high doses of phenytoin (500 ppm) treatment in a rodent model (Diwan, Henneman, & Nims, 2001). In addition, hepatic CYP2B‐mediated benzyloxyresorufin *O*‐dealkylase activity was dose‐dependently increased in the presence of phenytoin (Diwan et al., 2001). Increased CYP2B activity is strongly associated with the ability of numerous xenobiotics to promote carcinogenesis. Thus, liver tumorigenesis may be highly correlated with hepatic CYP2B activity. By contrast, a low dose of phenytoin was found to be nongenotoxic in a battery of cytogenetic assays and in an in vivo study (Jang et al., 1987; Kindig, Garriott, Parton, Brunny, & Beyers, 1992). Consistent with our findings, higher doses of phenytoin (>60 DDDs) were highly correlated with the risk of HCC; however, this association was not found in DDD of ≤60.

Our study has two limitations. First, the NHIRD does not provide patient details that might indicate confounding risk factors for HCC, such as body mass index, lifestyle factors (including smoking, alcohol consumption, and physical activity), environmental exposure, and family history of malignancy, which may play a critical role in the final outcome. Second, we were unable to contact the patients directly to obtain additional information because of the anonymized nature of the database. The claims data in the NHIRD were used primarily for administrative billing purposes and were not analyzed for scientific reliability for clinical studies.

## 5 Conclusion

We have found an association between higher doses of phenytoin and HCC, after having adjusted for phenobarbital and primidone, clonazepam, clorazepate and diazepam, and other AEDs, and for the comorbidities. This association was not confirmed for low‐dose phenytoin.

## Funding Information

This work was supported by Ministry of Science and Technology, Taiwan, R.O.C. (MOST105‐2320‐B‐039‐031), China Medical University Hospital, Taichung, Taiwan (DMR‐105‐050), in part by Taiwan Ministry of Health and Welfare Clinical Trial and Research Center of Excellence (MOHW105‐TDU‐B‐212‐133019), and “The Aim for The Top University Plan” from Traditional Chinese Medicine Research Center of China Medical University, Taichung, Taiwan. The funders had no role in study design, data collection and analysis, decision to publish, or preparation of the manuscript. No additional external funding received for this study.

## Conflicts of Interests

All authors declare that they have no conflict of interest.
